# The Diagnostic Accuracy of Serologic and Molecular Methods for Detecting Visceral Leishmaniasis in HIV Infected Patients: Meta-Analysis

**DOI:** 10.1371/journal.pntd.0001665

**Published:** 2012-05-29

**Authors:** Gláucia Fernandes Cota, Marcos Roberto de Sousa, Fábio Nogueira Demarqui, Ana Rabello

**Affiliations:** 1 Laboratory of Clinical Research, Centro de Pesquisas René Rachou, Fundação Oswaldo Cruz, Fiocruz, Belo Horizonte, Minas Gerais, Brazil; 2 Hospital Eduardo de Menezes, Fundação Hospitalar do Estado de Minas Gerais-FHEMIG, Belo Horizonte, Minas Gerais, Brazil; 3 Post-Graduate Program in Adult Health Sciences, Universidade Federal de Minas Gerais, Belo Horizonte, Minas Gerais, Brazil; 4 Statistic Department, Exact Sciences Institute, Universidade Federal de Minas Gerais, Belo Horizonte, Minas Gerais, Brazil; Institute of Tropical Medicine, Belgium

## Abstract

**Background:**

Human visceral leishmaniasis (VL), a potentially fatal disease, has emerged as an important opportunistic condition in HIV infected patients. In immunocompromised patients, serological investigation is considered not an accurate diagnostic method for VL diagnosis and molecular techniques seem especially promising.

**Objective:**

This work is a comprehensive systematic review and meta-analysis to evaluate the accuracy of serologic and molecular tests for VL diagnosis specifically in HIV-infected patients.

**Methods:**

Two independent reviewers searched PubMed and LILACS databases. The quality of studies was assessed by QUADAS score. Sensitivity and specificity were pooled separately and compared with overall accuracy measures: diagnostic odds ratio (DOR) and symmetric summary receiver operating characteristic (sROC).

**Results:**

Thirty three studies recruiting 1,489 patients were included. The following tests were evaluated: Immunofluorescence Antibody Test (IFAT), Enzyme linked immunosorbent assay (ELISA), immunoblotting (Blot), direct agglutination test (DAT) and polimerase chain reaction (PCR) in whole blood and bone marrow. Most studies were carried out in Europe. Serological tests varied widely in performance, but with overall limited sensitivity. IFAT had poor sensitivity ranging from 11% to 82%. DOR (95% confidence interval) was higher for DAT 36.01 (9.95–130.29) and Blot 27.51 (9.27–81.66) than for IFAT 7.43 (3.08–1791) and ELISA 3.06 (0.71–13.10). PCR in whole blood had the highest DOR: 400.35 (58.47–2741.42). The accuracy of PCR based on Q-point was 0.95; 95%CI 0.92–0.97, which means good overall performance.

**Conclusion:**

Based mainly on evidence gained by infection with *Leishmania infantum chagasi*, serological tests should not be used to rule out a diagnosis of VL among the HIV-infected, but a positive test at even low titers has diagnostic value when combined with the clinical case definition. Considering the available evidence, tests based on DNA detection are highly sensitive and may contribute to a diagnostic workup.

## Author Summary

Human visceral leishmaniasis (VL), a potentially fatal disease, has emerged as an important opportunistic condition in HIV infected patients. In immunocompromised patients, serological investigation is considered not an accurate diagnostic method for VL diagnosis and molecular techniques seem especially promising. Demonstration of Leishmania parasites in bone marrow aspirate or in other biologic specimen, either by visualization or culture, remains the most reliable diagnostic technique in the setting of HIV co-infection. However, these tests are difficult to perform in rural areas and some of them are invasive and carry a risk of complication. This work is a systematic review to evaluate the accuracy of serologic and molecular tests for VL diagnosis in HIV-infected patients. Two reviewers searched the literature, evaluating quality of studies and comparing performance of diagnostic tests. Thirty three studies were included. Most studies were carried out in Europe. Serological tests varied in performance, but with overall limited sensitivity. Based on the evidence, serological tests should not be used to rule out a diagnosis of VL among HIV-patients, but a positive test at even low titers has diagnostic value when combined with the clinical case definition. Tests based on DNA detection are highly sensitive and may contribute to a diagnostic workup.

## Introduction

Leishmaniasis gained higher clinical importance in individuals infected with HIV-1 (human immunodeficiency virus type-1) as an opportunistic infection in areas where both infections are endemic. In immunocompromised patients, the clinical course of the disease is even less specific and can be masked by other associated opportunistic infection [1]. Co-infected patients classically present a chronic clinical course and high rate of treatment failure [2]. There is no doubt that the actual number of documented cases of co-infection is underestimated due to the various problems in recognition, diagnosis and reporting of either HIV-1 infection, or leishmaniasis or both, in the setting of developing countries [3].

Parasitological diagnosis remains the gold standard in the diagnosis of leishmaniasis mainly because of its high specificity [4]. Demonstration of *Leishmania* parasites in bone marrow aspirate or in other biologic specimens, either by visualization or culture, is also the most reliable diagnostic technique in the setting of HIV co-infection. However, microscopic examination requires invasive procedures and *in vitro* parasite isolation is difficult and time-consuming.

Antileishmanial antibodies have high diagnostic value in immunocompetent patients [5], [6] and a wide range of serological methods varying in sensitivity and specificity are available for the VL diagnosis. For immunosupressed individuals, serological investigation is considered not an accurate diagnostic method since a large number of these patients do not harbor antibodies detectable by standard techniques based on studies done in Europe [7]–[9] and in Africa (6). Moreover, there is some doubt whether one serological technique would be superior to the other for the VL diagnosis among HIV-infected patients [8], [10]–[12] and if there is difference in tests performance among global regions.

Over the past 10 years, several molecular techniques targeting various parasite genes have been developed for VL diagnosis. The polymerase chain reaction (PCR) based method is the most common molecular test successfully used and its use looks specially promising in immunosupressed patients [13]–[16]. This technique has emerged as a more rapid, sensitive, and specific than the traditional diagnostic methods for VL diagnosis [15], [17], [18].

To our knowledge, antibody detection and molecular tests for the VL diagnosis among HIV-infected patients has not been systematically reviewed and synthesized. We therefore conducted a systematic review to summarize the evidence on diagnostic accuracy (sensitivity and specificity, likelihood ratio, diagnostic odds ratio and Q point from summary ROC curve) of available serological and PCR-based tests, according to the guidelines and methods proposed for diagnostic systematic reviews and meta-analysis [19], [20].The aim of this study is to appraise the diagnostic accuracy of serologic and molecular tests for detecting symptomatic visceral leishmaniasis in patients infected by HIV.

## Materials and Methods

### Literature Review

**Figure 1:**
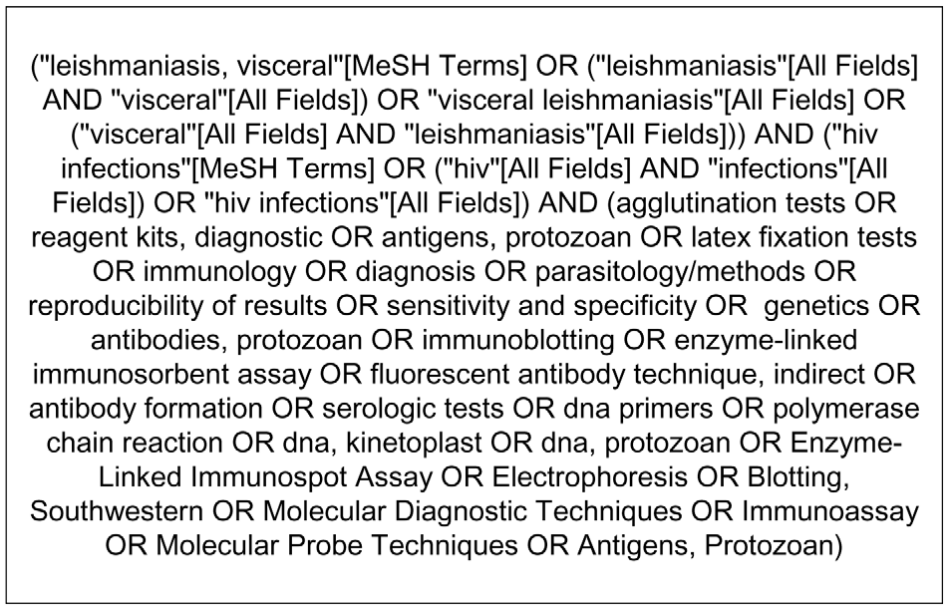
Terms used in PubMed search.

Selection was made independently by two reviewers (GFC and MRS) and discrepancies were solved by consensus after discussion. PubMed database search was performed using terms shown in Figure 1. A similar search by using Boolean operators in LILACS database was done.

The selected articles were read in full to confirm eligibility and doubts or disagreements were solved by discussion with a third author (AR). We searched both databases for articles published until 27, July 2011 that reported any available serologic or molecular tests for visceral leishmaniasis diagnosis in HIV-infected individuals over 14 years with symptomatic VL and diagnostic confirmation by examination by parasitological, serologic or molecular tests. No restrictions were made with respect to study design (cross sectional or case control) or data collection (prospective or retrospective). We obtained additional articles by citation tracking of review articles and original articles.

We excluded studies reporting other immune-depressing conditions when co-infected patients with HIV were not identified, series presenting 10 or less patients tested by the index test, review of series of cases and studies where separated results for each serologic test were not presented.

### Data Extraction

Data were extracted by one reviewer directly from the full length articles to structured tables containing all the descriptive variables and test results. A second researcher independently double checked the extraction of primary data from every study. Discrepancies were resolved by discussion. The following information was extracted: country in which the study was carried out, diagnostic methods applied, reference test used, characteristics of the participants, study design and quality, sample size, manufacturers and antigens used and titles for defining test positivity and outcome data (sensitivity and specificity were calculated when available data were presented). In many articles the numbers of true positive, false negative, true negative, and false positive observations were available. If not, we derived the numbers from the marginal totals and the reported sensitivity and specificity.

The number and type of participants were recorded and categorized as confirmed cases (HIV-infected individuals with VL) or controls (HIV-infected individuals without VL). Although some authors compared performance of tests in several different groups without VL, we selected only two possibilities of comparison group (control participants): 1- HIV-infected patients with the same clinical syndrome as confirmed cases with visceral leishmaniasis ruled out 2- HIV-infected patients without signs or symptoms of leishmaniasis.

### Assessment of Study Quality

We assessed the quality of studies using the Quality Assessment of Studies of Diagnostic Accuracy Approach-QUADAS [21], which contains 14 items specifically developed to assess the quality of primary studies of diagnostic tests.

### Data Synthesis and Statistical Analysis

The statistical analysis was based on the following steps: (1) qualitative description of findings; (2) search for the presence of publication bias, heterogeneity and threshold effect; (3) exploring possible explanations for heterogeneity; (4) statistical pooling of sensitivity, specificity and two global measures of accuracy of tests: diagnostic odds ratio (DOR) and symmetric summary receiver operating characteristic (sROC).

Publication bias was evaluated through Egger's test [22] by using Comprehensive Meta Analysis Software® v. 2.2.048 (CMA). Publication bias has been defined as the tendency on the part of investigators to submit, or the reviewers and editors, to accept manuscripts based on the direction or strength of the study findings. This definition concentrates on the fact that the strongest and most positive studies are most likely to be published.

Heterogeneity was explored with I^2^ estimate from Cochran Q (the most commonly used heterogeneity statistic) according to the formula: I^2^ = 100%×(Cochran *Q* –degrees of freedom)/Cochran *Q* [23]. One must understand heterogeneity as a greater variation of sensitivity, specificity or DOR between the included studies than is compatible with the play of chance. This statistical heterogeneity should represent other sources of differences such as clinical, tests or research design characteristics.

In nearly all situations sensitivity and specificity are not independent, what is called threshold effect. For this reason, sensitivity and specificity are considered inappropriate for meta-analyses, as they do not behave independently when they are pooled from various primary studies to generate separate averages [24]. The threshold effect may be caused by explicit differences in either positive cut-off definitions or implicit population and methodological differences among studies [19]. A robust approach to combining data and estimating the underlying relationship between sensitivity and specificity is the construction of a sROC curve. Methods that involve pooling sensitivities and specificities from individual studies, or combining positive and negative likelihood ratios fail to account for the paired nature of the parameters, and should generally be avoided [25].

According to Centre for Reviews and Dissemination (CRD) guidance for undertaking systematic reviews “where only one parameter (e.g. sensitivity, but not specificity) is presented, simple pooling of proportions is the only option. Assessment of single parameters is usually inappropriate, but is sometimes used when there is a specific clinical reason why only one parameter should be the focus of interest” [26]. Thus, given the small number of available studies and the paucity of data on the performance of the test in control populations (HIV-infected patients without VL), besides global analysis including few studies presented both sensitivity and specificity, we decided to pool sensitivity and specificity separately of all studies in order to compare results and check if both approaches would reach the same or different conclusions. Our intention was to discuss the methodological possibilities and assess the reliability of our results. Statistical analyses were carried out with the open source statistical language and environment R 2.0.1 [27].

To calculate sensitivity and specificity values for the tests, we cross-tabulated each result against the reference standard. Whenever possible, we extracted raw data from primary studies to fill in the four cell values of a diagnostic 2×2 table: true positives, false positives, true negatives, and false negatives. When studies did not provide confidence intervals for sensitivity or specificity, we estimated them from the reported 2×2 table [28] using Wilson score method [29].

When available, study results were pooled using a DerSimonian Laird method (random effects meta-analysis model) from Meta-Disc® 1.4 analysis software [30]. It was used to obtain pooled results of sensitivities, specificities, positive (PLR) and negative likelihood ratio (NLR). The likelihood ratio for a positive result is sensitivity divided by 1- specificity and tells how much the odds of the disease increase when a test is positive. A PLR can be used to assess the impact on diagnosis of a positive test result for an individual. The likelihood ratio for a negative result is 1- sensitivity divided by specificity and tells how much the odds of the disease decrease when a test is negative. Pooled likelihood ratio is useful since it can be used directly in the Bayes rule: Post-test odds = pre-test odds×LR. In addition, true positive rates (TPR = sensitivity) and false positive rates (FPR = 1-specificity) were summarized using a sROC curve [32]. The Q-point (point on curve where sensitivity equals specificity) obtained from the sROC curve was used as a measure of global accuracy [25]. Also used to compare overall accuracy among tests, diagnostic odds ratio (DOR) with fixed effects model were obtained from CMA® software. The DOR of a test is the ratio of PLR divided by NLR. Pooling sensitivity and specificity separately assumes that the diagnostic threshold is the same in each study. Pooling DORs relaxes this assumption by assuming that the studies relate to the same sROC curve. The DOR has been put forward as a useful single indicator of test performance, which indicates the strength of the association between test results in disease [31]. It is difficult to be clinically interpreted, but useful from the statistical point of view in the assessment of the overall test accuracy in meta-analysis [19], [31], [33].

## Results

**Figure 2:**
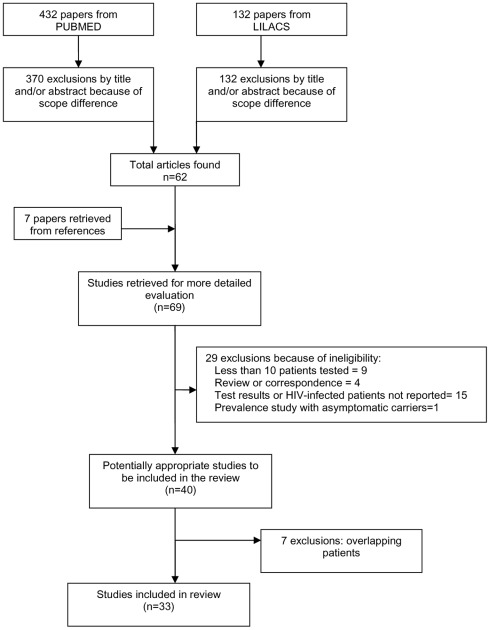
Study selection process.

From the literature searches, we identified 432 primary citations from PubMed and 132 from LILACS. Seven additional articles (references from primary articles) were also found. Publication year ranged from 1989 to 2009. Study selection flow is shown in Figure 2.

All 132 citations from LILACS and 370 from PubMed were excluded by the reading of titles and/or abstracts. Thirty three more articles were excluded after reading the entire article: nine reported less than 10 patients tested, four were a review or correspondence, fifteen studies did not report tests results or the immunosupressed patients could not be identified, one was a prevalence study to detect the presence of asymptomatic carriers in a given population and seven studies were excluded because they included cases published elsewhere. Thirty three studies recruiting 1489 patients were included. A total of six different serological tests were found: direct agglutination test (DAT), indirect fluorescent antibody test (IFAT), Enzyme linked immunosorbent assay (ELISA), Immunoblotting (Blot), rapid K39-based immunochromatographic test and Haemagglutination (HA). We found only two studies [34], [35] addressing the performance of two different commercial counter-immunoeletrophoresis tests (commonly referred to as rapid diagnostic tests) among HIV-infected patients. Only one from them stated sensitivity and specificity. No studies involved individuals younger than 15 years old. Table S1 summarizes the characteristics of the 33 studies.

The study quality analysis as assessed by QUADAS tool showed that 24 out of 33 studies (73%) met more than seven criteria (Table S2). Regarding study design and execution, fifteen studies were identified as retrospective or a clinical database analysis (set of data systematically gathered on all patients even though no specific analysis was prospectively planned). Sixteen reports were truly prospective and two had transversal design. In addition, a minority of them (8 studies) reported consecutive patient inclusion as the method of participant selection. Only two studies [8], [36] reported at least single blinded interpretation of index test and reference standard results. For most studies information about the condition of the specimens (frozen or fresh) was unclear or not reported. Three studies [12], [37], [38] reported that antibody detection was done with stored sera.

The reference standard for all studies was a positive result on direct microscopically examination or culture of blood or bone marrow aspirate, and in few cases, from another sample tissue. In one study diagnosis could also be confirmed by serology [16] or detection of parasites by polymerase chain reaction [39] associated with clinical signs. In these two studies the index test did form part of the reference standard. In 7 of 13 studies evaluating control patients, the entire study population was investigated using the identical reference standard (complete verification). In other five studies the reference standard for VL patients and control participants differed (e.g., parasitological tests for VL patients and serological tests for control participants (differential verification) and one study did not report the test used for control verification [35]. Five studies had as control group HIV-infected individuals without clinical signs of disease.

Most studies (28/33) included less than 100 patients and only 14 out 33 studies (42%) provided detailed clinical characterization of the studied population. The specific antigen composition was described in 14 out of 21 studies (62%) evaluating IFAT tests. Six from these studies used a commercial test based on axenic cultures of *L. infantum*; two other studies used commercial tests based on *L. tropica* [40] and *L. donovani* [41] culture. Seven studies used antigen prepared from whole promastigotes of the World Health Organization strain.

Published experience with ELISA is very scarce: there are six studies. Two studies searched antibodies to recombinant (r) antigen K39 while other two [18], [42] used antigen extracted from promastigotas strain of *L. infantum*. The other two studies [43], [44] assessing ELISA performance did not report the antigen used.

Regarding immunoblotting, most authors considered the criterion for positivity the detection of antibodies to the 14- kD antigens with or without antibodies to other low molecular weight bands [18], [43]–[46]. Santos-Gomes and others [10] assumed as positive result the presence of any band since the sera from the control groups did not recognize any *Leishmania* antigen. Medrano et al. [8] considered an immunoblotting reactive when one or more bands of any molecular weight detected were present in at least two patients with VL, but not in the negative control sera from the no endemic area.

Details of the PCR techniques used are summarized in Table S1. Whole blood was used in all but four studies evaluated also PCR use in bone marrow samples [16], [39], [47], [48]. Several variations in the PCR technique were used: small subunit ribosomal RNA (ssu-RNA) from *L. infantum* [16], [39], [48]–[50], ssu-rRNA from *L. donovani* [36], repetitive nuclear sequence (140 bp) from *L. infantum* [18] and nested-PCR (100 bp) from *L. infantum*.

Only 13 studies evaluating 5 tests [7], [8], [10], [12], [35], [36], [44], [46], [50]–[54] showed results of both sensitivity and specificity, the requirement to testing threshold effect presence. It should be noted however that for samples with less than 10 studies is not possible to state at significance level of 5% there is not threshold effect for studies in which the correlation result was negative.

**Figure 3:**
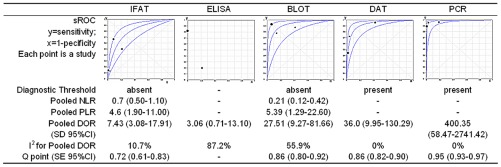
Tests performance summary. Footnote: Immunofluorescence antibody test (IFAT), Enzyme linked immunosorbent assay (ELISA), Immunoblotting (BLOT), direct agglutination test (DAT) and polymerase chain reaction (PCR) in whole blood, standard deviation (SD), 95% confidence interval (95% CI), *standard error (SE). * SE is a measure of precision and it is not a measure of confidence interval, which is shown in sROC plot, except for ELISA.

Corresponding sROC plots of the studies and estimated DOR (95% confidence interval) of tests are shown in Figure 3. For DOR analysis, the global accuracy of DAT 36.01 (9.95–130.29, I^2^ = 0) and Blot 27.51 (9.27–81.66, I^2^ = 56) was comparable and higher than IFAT 7.43 (3.08–1791, I^2^ = 11) and ELISA 3.06 (0.71–13.10, I^2^ = 87), in spite of wide confidence intervals. PCR in whole blood had the highest DOR: 400.35 (58.47–2741.42, I^2^ = 0). Pooled NLR for IFAT 0.7 (0.5–1.1) is higher than Blot 0.21 (0.12–0.42). Egger's test 2-sided p value was larger than 0.05, suggesting absence of publication bias for all tests.

**Figure 4:**
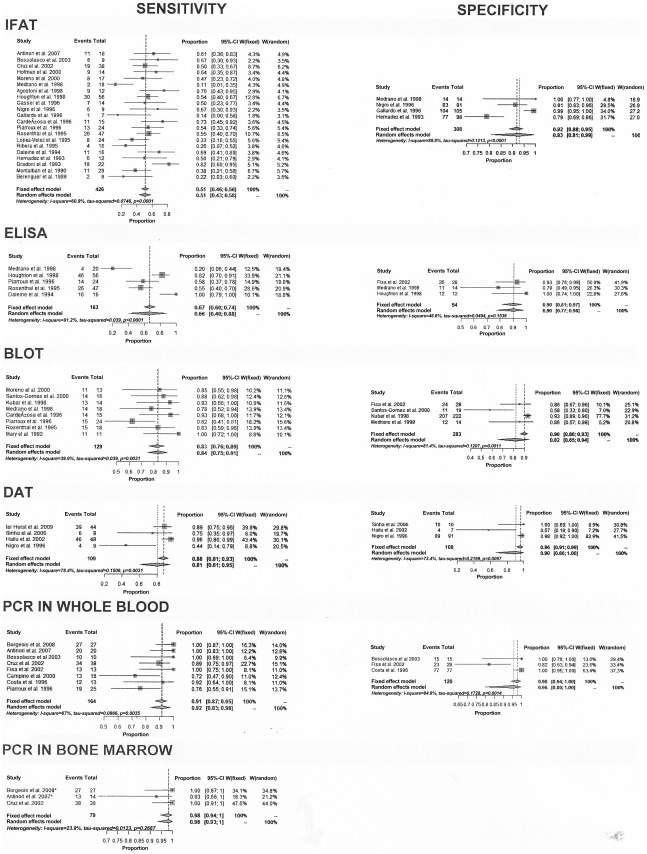
Estimates of sensitivity and specificity (95% confidence interval) of tests. Footnote: Combined results are shown using both options: fixed and random effects model. When both results are similar with low heterogeneity, both can be used. When they are different, we prefer results from random effects model, which gives wide and conservative confidence interval for heterogeneous results.

Figures 4 show the results of individual and combined sensitivity and sensitivity estimates for the tests including all studies. On the whole, sensitivity varied widely among studies of a given type of test and in studies across different types of tests. There is high heterogeneity across studies for most tests (Table S3 and Table S4). Although we used random effects model to summarize data, a point estimate of separated sensitivity or specificity must be evaluated carefully. IFAT was the test most frequently evaluated in the review (21 studies) with sensitivity values ranged from 11% to 82%. Sensitivity was less than 50% in ten out 21 (48%) studies; specificity value ranged from 79% to 100%, with specificity >90% in three out of four (75%) studies. The estimated sensitivity for the IFAT using random effects model was 51% (95% confidence interval 43% to 58%). Three studies [8], [40], [53] had even lower sensitivity (11, 14 and 22%). Although all three had used as cutoff 1∶80, we carried out separate analyses in subgroups stratified by cut-off value, sample size, study design, geographical region, and type of controls and we did not find any significant difference except for QUADAS score, which showed an inverse association with sensitivity (data not shown). It was not possible to assess the heterogeneity between studies according to geographic region due to the small number of studies outside Europe.

The estimated sensitivities using random effects model and their respective 95% confidence intervals for the other tests were: Blot 84% (75% to 91%), DAT 81% (61% to 95%), ELISA 66% (40% to 88%), PCR in whole blood 92% (83% to 98%) and PCR in bone marrow 98% (93% to 100%). Regarding specificity, we also found significant heterogeneity for the same test across several studies but high overall pooled specificity for all of them. The estimated specificity using random effects model and their respective confidence intervals for following tests were: Blot 82% (65% to 94%), ELISA 90% (77% to 98%), IFAT 93% (81% to 99%), DAT 90% (66% to 100%), PCR in whole blood 96% (80 to 100%).

**Figure 5:**
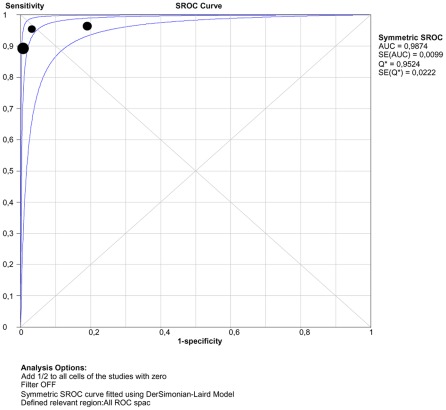
sROC curve for PCR in peripheral whole blood.

Figure 5 shows performance for PCR in peripheral blood through a sROC curve. The accuracy of PCR based on Q-point was 0.95; 95%CI 0.92–0.97, which means good overall performance.

## Discussion

Our data allow some conclusions based on available evidence, which essentially reflect the European experience with serological and molecular diagnosis of VL among HIV-infected: (1) the available evidence is limited and there is great variability among the studies; (2) the accuracy of molecular methods is greater than the serological methods; (3) DAT and Blot have better global accuracy among serological tests; (4) although specificity was generally high for all serological tests, there is unexpectedly high variation in specificity among studies evaluating the same test; (5) serological tests vary widely in performance, but with overall limited sensitivity in HIV infected patients. It is very important to note that high concentration of cases of *Leishmania*-HIV co-infection is found in Africa and Asia continents and it is possible that these findings can not be extrapolated to these populations. However, this is a critical summary of the evidence currently available.

Several indicators of diagnostic performance have been proposed, such as sensitivity and specificity. Using paired indicators can be a disadvantage in comparing the performance of competing tests, especially if one test does not outperform the other on both indicators. The DOR is a single indicator of diagnostic performance; it facilitates formal meta-analysis of studies on diagnostic test performance [31]. Based on DOR, we observed superiority of PCR above serologic methods. Among serologic tests, based on DOR, we observed that Blot and DAT are superior to ELISA and IFAT. Pooled NLR (95% confidence interval) for IFAT 0.74 (0.51–1.09) is higher than Blot 0.21 (0.12–0.42), confirming that especially IFAT is not an appropriate test to exclude diagnosis, since its negative predictive value will be low. Blot and DAT had better sensitivities than the other serologic tests evaluated.

The sensitivity values of each study are consistent with the values of the studies combined and we can affirm that IFAT sensitivity is very limited and heterogeneous. An explanation for this heterogeneity is the quality of the studies, which is suggested by the indirect comparison between QUADAS score and sensitivity of IFAT. Among the studies assessing IFAT, one study [37] is distinguished by exceptionally high sensitivity displayed (81%). According to this author, the performance obtained using IFAT prepared with reference *L. infantum* strain from WHO (MHOM/TN/80/IPT1) was significantly better than prior local experience with commercial IFAT kits. This may mean that variables related to the preparation of the antigen and regional differences in prevalence and strain of *Leishmania*, in addition to the characteristics of the tested populations, have a greater impact on test performance [55].

Pooled specificity is high among all serologic tests. Nevertheless, we found great variability in the results for the same test across different studies. Especially for one DAT study [54] and one Blot study [10], the specificity results were very low, associated with high sensitivities, suggesting threshold effect. The sensitivity and specificity of such diagnostic methods depend on the type, source, and purity of antigen employed, as some of the *Leishmania* antigens have common cross-reactive epitopes shared with other microorganisms such *Trypanosome brucei* subspecies, *Trypanosome cruzi* and *M. tuberculosis* [6]. In addition, the type of controls significantly influenced the estimates for specificity. Studies including healthy controls tend to show higher specificity than those recruiting patients with clinically suspected disease consecutively and prospectively in a representative clinical setting.

DAT based on whole promastigotes of *L. donovani* or *L. infantum* are tests used widely for the diagnosis of VL. However, the major disadvantage of this technique is the limited production facility of quality controlled antigen. A recent meta-analysis of the DAT performance among immunocompetent individuals showed sensitivity and specificity estimates (and 95% confidence interval) of 94.8% (92.7% to 96.4%) and 97.1% (93.9% to 98.7%), respectively [56]. Despite lower performance in HIV-infected patients than in immuncompentents, DAT (and Blot) proved to be the most effective serological technique in those immunosupressed by HIV infection. However, only four studies assessing DAT with sensitivities and specificities, none from Latin-America and only one from Europe could be included. One [7] out of these four studies exhibited discrepant and very low sensitivity, despite use of 1∶400 cut-off. Specifically this study was performed in Italy (the others were conducted in India and Ethiopia) and DAT was carried out using promastigotes of *L donovani sensu latu*. This may represent the relevance of the prevalent strain in the performance of a test prepared from promastigotes (local antigen specificity) or the difference in immune response induced by more or less anthroponotic strains of *Leishmania*.

Some heterogeneity in sensitivity of the tests seemed to be related to the geographical location of the study. Differences in test performance between regions is attributable basically to parasite diversity [57], but it can also be related to differences in antibody concentrations which may in turn be linked to different age patterns, immune and/or nutritional status of patient. In this review, it was not possible to evaluate the test's performances in various endemic regions of world. All included studies assessing IFAT, Blot and ELISA were performed in Europe. Data on DAT essentially reflect the response in Ethiopia and Italy, with only one study performed in India (few patients). No study from the Americas was found. Regarding rK39 dipstick test, the only two studies [34], [35] found, one carried out in India and one in Ethiopia, exhibited different sensitivities. Similarly, among non HIV-infected patients results between global regions were substantially different. There is data showing the low sensitivity of rK39 based dipsticks in Sudan [58]–[60] and better results in studies from South Asia [56].

Serological tests have an already recognized low sensitivity for the diagnosis of VL among HIV-patients [61]. Gradoni et al. [37] suggested that the serological response could be related to the sequence of temporal acquisition of the infectious agents. Seropositivity would represent a reactivation of latent infection before the immune depression caused by the viral infection (asymptomatic carriers), while seronegativity would result from primary *Leishmania* infections after viral infection. However, the severe dysfunction of T and B lymphocytes in HIV-infected individuals, an alteration in antigen presentation by macrophages or in T and B lymphocyte cooperation would explain the decrease in specific antibody production, as occurs for other infections [1]. It is also necessary to note that most serological studies from Europe date from the early stages of the HIV epidemic, while the PCR studies were usually done when HAART was available. Possibly, different types of populations (more advanced HIV disease in the earlier studies) were included.

Standard techniques for assessing diagnostic tests assume that a definitive reference test is available, that is, that the reference test used is as close to 100% accurate as can be. However, it may be either that the available test is far from perfect or that such a test simply does not exist [23]. The presence of *Leishmania* parasites may only be demonstrated incontrovertibly by the microscopically examination of smears or the culture of blood or biopsy samples. Microscopical examination of spleen aspirates is sensitive and specific but requires expertise to carry out the aspiration safely and to read the slides accurately. Examination of bone marrow or lymph node aspirates is equally specific but less sensitive [62]. Parasite load is quite heavy in VL-HIV co-infected patients and the presence of *Leishmania* amastigotes in the bone marrow can often be demonstrated. However there are well-described instances in the literature where amastigotes were not demonstrable in bone marrow, though they were found at unexpected locations like the stomach, the colon, or the lungs [5]. The majority (31/33, 94%) of studies used exclusively microscopic determination of parasites as the reference standard. Although direct and culture are considered the definitive diagnosis, parasitological methods does not detect all cases of VL; therefore, some degree of misclassification of disease for study participants was possible.

In the case of molecular tests, previous studies suggest their greater sensitivity compared to the classical parasitological methods [13]. As a result, some authors used a combination of several laboratory tests and clinical manifestations as reference test. Incorporation bias occurs where the experimental test is used as part of the reference strategy, that is, the experimental test and reference tests are not independent, leading to overestimation of both sensitivity and specificity. Based on prevalence studies, the proportion of individuals identified as asymptomatic carriers of *Leishmania* by PCR methods is not negligible [63]. None of the studies testing PCR included here assessed the proportion of asymptomatic patients co-infected with *Leishmania* and HIV. An important point to notice is that molecular tests are still expensive and require sophisticated laboratory setting; these features represent real obstacles to their implementation in the regions with the highest absolute numbers of HIV-VL coinfection cases (East-Africa and India). This performance data can be used to guide priority setting for field trials and/or procurement decisions. The final decision on product selection needs to be taken in a rational way, considering not only the minimal performance limits, but also the global endemic region, patient characteristics, experience of the intended users, climate and costs.

During the past few years, numerous studies have investigated *Leishmania* antigen expressions at the level of specific antibody recognition. Using immunoblotting techniques, several *L. infantum* antigens that appeared promising for establishing an immunodiagnosis of VL in nonimmunocompromised hosts have been identified [8]: 70–72 kD, 94 kD, 14–16 kD, 39 kD, 24 and 32 kD, but a clear pattern of specific immune response to parasite antigens during the active course of the disease has not been yet defined. Mary and others [64] in a series of 11 AIDS cases found a similar pattern of reactivity between HIV and non-HIV patients with VL that differed only in the variable presence of a 14-kD band in the former group. The 16-kD antigenic component was considered as the more sensitive and specific diagnostic band. Rosenthal and others [43] in another study carried out in the same endemic area (southern France) reported the presence of bands of molecular mass 14 kD and 16 kD in 15 of the 18 evaluated cases. In Medrano et al [8] study, immunoblots were found to be reactive during the active course of the disease in 78% of the cases. Five groups of parasite antigens (14 kD, 42–43 kD, 57 kD, 76 kD, and 94 kD) appear to have potential use for diagnosis although the pattern of reactivity observed during the acute VL disease was very variable. Among the nine studies evaluating immunoblotting, performance described by Piarroux and colleagues [18] is distinguished by low sensitivity (63%). In this study, unlike the others, a more sensitive reference test was used and included visualization of *Leishmania* in any specimen collected at the same period, besides in bone marrow aspirate. However, strict comparisons between results reported in the literature are rather difficult because of the variability in the techniques and the use of different strains and antigens. Considering the high variability of the immunoblotting patterns, it seems that a combination of several antigens should be used, as has been previously suggested [8], [65].

Moreover, different settings (e.g., difference in *Leishmania* prevalence may have accounted for some variation in test performance. It is often assumed that indices of test accuracy such as sensitivity and specificity are fixed (for a given threshold). But they can vary as a function of prevalence [66], [67]. When spectrum bias is present, either sensitivity or specificity would be expected to change. Sensitivity would be expected to increase where test results become more extreme in patients with the most severe disease (i.e. more likely to test positive). Specificity is affected by a variety of alternative diagnosis in those without the target disorder that could cause false positive results. The range of such diagnosis is likely to be wider in studies that have a lower prevalence of the target disorder [23]. Another problem concerned to limited information on clinical status and disease severity in the populations tested. Differing criteria for patient selection, age, duration of illness and severity of HIV-disease of the study populations may have introduced significant variability in findings among studies (selection bias).

There are also limitations in studies methods. The differences in PCR methods included the nature of the samples (whole blood or bone marrow aspirate), volume tested, DNA extraction procedures, choice of target gene, detection of PCR products and the use of appropriate controls. All of these factors have been reported as likely causes of heterogeneity and they were all present in the studies included. Interpretation of many diagnostic tests involves some degree of subjective interpretation. Only two of the studies (6%) reported blinded interpretation of the results of the index test and the reference standard. Lack of blinding may have resulted in an overestimation of the sensitivity of the index test result. In additional, the condition of specimens may also have affected the sensitivity results. The vast majority (91%) of studies did not report if frozen or fresh sera were used. In 5 from 13 studies (38.5%), different diagnostic tests were performed in VL patients and control participants: parasitological tests for patients and serological test for control participants [verification bias]. In one study [35] the information about the test used to rule out VL was not reported.

This comprehensive review is mainly limited by quality of available studies. We believe that pooled measures from different studies help to appraise global accuracy. Nevertheless, its validity remains on scarce evidence that may change as larger well designed studies are done. No large prospective clinical studies evaluating either serological or molecular tests have been reported. In addition, available data are not representative of all endemic regions. Data about HA, rapid K39-based immunochromatographic and PCR test in bone marrow aspirate could not be analyzed due to the paucity of studies. The same way, there were too few studies to explore by subgroup analysis or metaregressing whether the diagnostic yield of the methods was different among subgroups (i.e., control characteristics, sample size, study design and quality) or whether the different techniques influenced the results. The heterogeneity among studies evaluating the same method makes pooled sensitivity and specificity measures less reliable. The strategies of pooling sensitivity and specificity or using global accuracy measures like DOR and sROC are subject to different kind of bias. Including studies with more quality, although in low number, reduce the bias of methodological flaws changing results. Including more studies pooling separately sensitivity and specificity reduce the bias of selecting low number of studies. In spite of these different biases, by using both methods we found very similar results confirming the consistency of these observations.

### Conclusions

The results of this evaluation confirm the low sensitivity of the serologic tests for VL diagnosis in HIV-infected patients. Except for DAT, currently available evidence about performance of serological tests refers to *Leishmania infantum chagasi*, the etiological agent of visceral leishmaniasis in the Americas and in the Mediterranean basin. Our results indicated superiority of Blot over IFAT and ELISA. DAT seems to be better than IFAT and ELISA, like Blot, but its performance may be influenced by difference in geographic region, meaning different *Leishmania* species. As the performance of DAT and Blot is comparable, the choice should be made on the basis of other criteria such as region, cost, feasibility, and sustainability. Given these findings, we express concern that IFAT remains the most frequently serological test used for VL investigation in South America, even among HIV-infected. At this time, there is no evidence to support recommendations on serologic or molecular diagnosis of VL in patients infected with HIV and living in East Africa or Southeast Asia. The development of the rK39 dipstick has brought a major improvement in the diagnosis of VL in non HIV-infected patients in the field. Nevertheless, the paucity of data about the rK39 dipstick in HIV-infected patients underscores the need for more research before it being integrated in a diagnostic algorithm.

In spike of lack of homogeneity of the PCR methods used, available evidence suggests that, at this point in time, published data on molecular tests produce consistently good estimates of accuracy. Its main weaknesses are the lack of standardization for the technique. We must also point out that the meaning of *Leishmania* infection detected by PCR in asymptomatic individuals is not yet defined. This fact might raise questions about possible false-positive results. In addition, alternative methods must be developed to solve the “gold standard problem”. A promising strategy is Bayesian latent class models [68].

More studies are needed to compare tests for VL diagnosis in different regions. This highlights the need to implement a diagnostic algorithm as appropriate for each global endemic area. The design of diagnostic studies must follow the STARD initiative [69] as a way to minimize bias.

In conclusion, based on the available evidence, serology should not be used to rule out a diagnosis of VL among HIV-infected patients. An additional molecular or parasitological test may be necessary if results of serological tests are negative. A positive serological test at even low titers has diagnostic value when combined with the clinical case definition. In its turn, tests based on PCR are highly sensitive and should contribute to the diagnosis, especially in areas of low endemicity.

## Supporting Information

Table S1 **The characteristics of studies.** Footnote: DiaMed IT-Leish (DiaMed AG, Switzerland) ‡ Kalazar Detect Rapid Test (In Bios International, Inc., Seattle, USA) PB: peripheral blood BMA: bone marrow aspirate bp: base pair n-PCR: nested PCR ssU-rRNA: small subunit ribosomal RNA NR: not reported NA: not applicable PCR: polymerase chain reaction # only new visceral leishmaniasis cases included, number of patients with relapses not reported.
(DOC)

Table S2 **QUADAS scoring for each study.** Footnote: If the answer is “no” or “unclear” = score 0 If the answer is “yes” (x) = score 1 QUADAS ITENS: 1. Was the spectrum of patients representative of the patients who will receive the test in practice? 2. Were selection criteria clearly described? 3. Is the reference standard likely to correctly classify the target condition? 4. Is the time period between reference standard and index test short enough to be reasonably sure that the target condition did not change between the two tests? 5. Did the whole sample or a random selection of the sample, receive verification using a reference standard of diagnosis? 6. Did patients receive the same reference standard regardless of the index test result? 7. Was the reference standard independent of the index test (i.e. the index test did not form part of the reference standard)? 8. Was the execution of the index test described in sufficient detail to permit replication of the test? 9. Was the execution of the reference standard described in sufficient detail to permit its replication? 10. Were the index test results interpreted without knowledge of the results of the reference standard? 11. Were the reference standard results interpreted without knowledge of the results of the index test? 12. Were the same clinical data available when test results were interpreted as would be available when the test is used in practice? 13. Were uninterpretable/intermediate test results reported? 14. Were withdrawals from the study explained?
(DOC)

Table S3 **Individual performance of studies evaluating serological tests.**
(DOC)

Table S4 **Individual performance of studies evaluating molecular tests.** Footnote: polimerase chain reaction (PCR).
(DOC)

Checklist S1 **PRISMA checklist.** Footnote: *From:* Moher D, Liberati A, Tetzlaff J, Altman DG, The PRISMA Group (2009). Preferred Reporting Items for Systematic Reviews and Meta-Analyses: The PRISMA Statement. PLoS Med 6(6): e1000097. doi:10.1371/journal.pmed1000097.
(DOC)
