# Supplementary material for: The Diagnostic Accuracy of Serologic and Molecular Methods for Detecting Visceral Leishmaniasis in HIV Infected Patients: Meta-Analysis
Source: PLoS Negl Trop Dis. 2012 May 29;6(5):e1665. doi: 10.1371/journal.pntd.0001665 (PMC3362615; doi:10.1371/journal.pntd.0001665)
Supplement: Table S1 — The characteristics of studies. Footnote: DiaMed IT-Leish (DiaMed AG, Switzerland) ‡ Kalazar Detect Rapid Test (In Bios International, Inc., Seattle, USA) PB: peripheral blood BMA: bone marrow aspirate bp: base pair n-PCR: nested PCR ssU-rRNA: small subunit ribosomal RNA NR: not reported NA: not applicable PCR: polymerase chain reaction # only new visceral leishmaniasis cases included, number of patients with relapses not reported. (DOC) [file pntd.0001665.s001.doc]

Table S1- The characteristics of studies

| **Reference** | **Country** | **Test index**  **IFAT and DAT (cut-off)**  **ELISA (antigen)**  **molecular test (type of PCR)**  **Blot (bands used as positive criteria)** | **Sample size**  **(VL-HIV/controls)** | **Study design** | **Reference test** | **Quadas**  **score** | **Controls**  **(HIV-Infected without VL)** |
| --- | --- | --- | --- | --- | --- | --- | --- |
| **ter Horst et al. 2009** | Ethiopia | DAT (1:3200) | 44/- | Prospective, comparative of tests | Parasitological | 9 | NA |
|  |  | K39rapid test: DiaMed-IT-Leish ® † |  |  |  |  |  |
| **Bourgeois et al. 2008** | France | PCR (PB and BMA): ssU-rRNA (603 bp): *L. infantum* | 27/- | Prospective | Parasitological and/or PCR | 7 | NA |
| **Antinori et al. 2007** | Italy | IFAT (1:40) | 20/- | Prospective, comparative of tests | Parasitological and/or serologic test | 7 | NA |
|  |  | PCR (PB and BMA): ssU-rRNA (359 bp): *L. infantum* |  |  |  |  |  |
| **Goswami et al. 2007** | India | K39rapid test: Kalazar Detect Rapid Test® ‡ | 12/60 | Prospective | Parasitological | 7 | Symptomatic HIV-patients without VL confirmation |
| **Sinha et al. 2006** | India | DAT (1:800) | 8/10 | Prospective | Parasitological | 6 | Asymptomatic HIV-patients |
| **Bossolasco et al. 2003** | Italy | IFAT (1:80) | 10/15 | Prospective | Parasitological | 10 | Symptomatic HIV-patients without VL confirmation |
|  |  | PCR (PB): ssU-rRNA real-time:*L. infantum* |  |  |  |  |  |
| **Cruz et al. 2002** | Spain | IFAT (1:80) | 38/- | Prospective | Parasitological | 12 | NA |
|  |  | PCR (PB and BMA): ssU-rRNA n-PCR (358 bp): *L. infantum* |  |  |  |  |  |
| **Fisa et al. 2002** | Spain | ELISA (NR) | 15/28 | Prospective | Parasitological | 10 | Symptomatic HIV-patients without VL confirmation |
|  |  | BLOT (bands 70, 65, 46, 30, 28, 14 or 12 kD) |  |  |  |  |  |
|  |  | PCR (PB): n-PCR (100 bp): *L. infantum* |  |  |  |  |  |
| **Hailu et al. 2002** | Ethiopia | DAT (1600) | 51/7 | Retrospective | Parasitological | 11 | Symptomatic HIV-patients without VL confirmation |
| **Campino et al. 2000** | Portugal | PCR (PB): ssU-rRNA (600 bp): *L. infantum* | 18/- | Retrospective | Parasitological | 8 | NA |
| **Moreno et al. 2000** | Spain | IFAT (1:80) | 17/- | Retrospective | Parasitological | 10 | NA |
|  |  | BLOT (any band) |  |  |  |  |  |
| **Hofman et al. 2000** | France | IFAT (1:80) | 16/- | Retrospective | Parasitological | 11 | NA |
| **Santos-Gomes et al. 2000** | Portugal | BLOT (at least one band) | 16/- | Prospective, comparative tests | Parasitological | 9 | Symptomatic HIV-patients without VL confirmation |
| **Medrano et al. 1998** | Spain | IFAT (1:80) | 20/14 | Retrospective | Parasitological | 11 | HIV-patients who died of a non-VL cause |
|  |  | ELISA (K39 antigen) |  |  |  |  |  |
|  |  | BLOT (any band) |  |  |  |  |  |
| **Houghton et al. 1998** | Italy | IFAT (1:80) | 56/12 | Transversal, comparative | Parasitological | 9 | Asymptomatic HIV-patients |
|  |  | ELISA (K39 antigen) |  |  |  |  |  |
| **Kubar et al. 1998** | France | BLOT (bands 14 or 18 kD) | 14/222 | Prospective | Parasitological | 9 | Asymptomatic HIV-patients |
| **Agostoni et al. 1998** | Italy | IFAT (1:40) | 22/- | Retrospective | Parasitological | 12 | NA |
| **Costa et al. 1996** | France | PCR (PB): ssu-Rrna (nr): *L. Donovani* | 13/77 | Prospective | Parasitological | 7 | Symptomatic HIV-patients without VL confirmation |
| **Gasser et al. 1996** | Spain | IFAT (1:40) | 19/- | Retrospective | Parasitological | 8 | NA |
| **Nigro et al. 1996** | Italy | IFAT (1:100) | 9/91 | Prospective | Parasitological | 8 | Asymptomatic HIV-patients |
|  |  | DAT (1:400) |  |  |  |  |  |
| **Piarroux et al. 1996 #** | Spain | IFAT (1:80) | 25/- | Prospective | Parasitological | 8 | NR |
|  |  | ELISA (promastigotes *L. infantum MCAN/FR/73/LPM 56)* |  |  |  |  |  |
|  |  | Blot (bands 14 or 16 kD) |  |  |  |  |  |
|  |  | PCR (PB): Repetitive nuclear sequence (140 bp): *L. infantum* |  |  |  |  |  |
| **Gallardo et al. 1996** | Spain | IFAT (1:80) | 7/105 | Transversal | Parasitological | 10 | Asymptomatic HIV-patients |
| **Cardeñosa et al. 1996** | Spain | IFAT (1:80)  BLOT | 15/- | Retrospective | Parasitological | 6 | NA |
| **Rosenthal et al. 1995** | France | IFAT (NR) | 50/- | Retrospective | Parasitological and/or serologic test | 11 | NA |
|  |  | ELISA (NR) |  |  |  |  |  |
|  |  | BLOT (bands 14 or 16 kD) |  |  |  |  |  |
| **Ribera et al. 1995** | Spain | IFAT (1:40) | 20/- | Retrospective | Parasitological | 12 | NA |
| **Lopez-Velez et al. 1995** | Spain | IFAT (1:80) | 25/- | Retrospective | Parasitological | 10 | NA |
| **Daleine et al. 1994** | France | IFAT (1:100) | 16/- | Retrospective | NR | 3 | NA |
|  |  | ELISA (promastigotes *L. infantum MON- 1 Biokema)* |  |  |  |  |  |
| **Hernandez et al. 1993** | Spain | IFAT (1:40) | 19/103 | Prospective | Parasitological | 6 | Symptomatic HIV-patients without VL confirmation |
| **Gradoni et al. 1993** | Italy | IFAT (1:80) | 22/- | Retrospective | Parasitological | 6 | NA |
| **Mary et al. 1992** | France | BLOT (bands 14 or 16 kD) | 11/- | Prospective | Parasitological | 10 | NA |
| **del Mar et al. 1991** | Spain | HA (1:60) | 12/- | Prospective | Parasitological | 9 | NA |
| **Montalban et al. 1990** | Spain | IFAT (1:40) | 40/- | Retrospective | Parasitological | 8 | NA |
|  |  | HA |  |  |  |  |  |
| **Berenguer et al. 1989** | Spain | IFAT (1:80) | 9/- | Retrospective | Parasitological | 10 | NA |
