# Supplementary material for: The Diagnostic Accuracy of Serologic and Molecular Methods for Detecting Visceral Leishmaniasis in HIV Infected Patients: Meta-Analysis
Source: PLoS Negl Trop Dis. 2012 May 29;6(5):e1665. doi: 10.1371/journal.pntd.0001665 (PMC3362615; doi:10.1371/journal.pntd.0001665)
Supplement: Table S2 — QUADAS scoring for each study. Footnote: If the answer is “no” or “unclear” = score 0 If the answer is “yes” (x) = score 1 QUADAS ITENS: 1. Was the spectrum of patients representative of the patients who will receive the test in practice? 2. Were selection criteria clearly described? 3. Is the reference standard likely to correctly classify the target condition? 4. Is the time period between reference standard and index test short enough to be reasonably sure that the target condition did not change between the two tests? 5. Did the whole sample or a random selection of the sample, receive verification using a reference standard of diagnosis? 6. Did patients receive the same reference standard regardless of the index test result? 7. Was the reference standard independent of the index test (i.e. the index test did not form part of the reference standard)? 8. Was the execution of the index test described in sufficient detail to permit replication of the test? 9. Was the execution of the reference standard described in sufficient detail to permit its replication? 10. Were the index test results interpreted without knowledge of the results of the reference standard? 11. Were the reference standard results interpreted without knowledge of the results of the index test? 12. Were the same clinical data available when test results were interpreted as would be available when the test is used in practice? 13. Were uninterpretable/intermediate test results reported? 14. Were withdrawals from the study explained? (DOC) [file pntd.0001665.s002.doc]

Table S2- QUADAS scoring for each study

| **QUADAS item**  **Reference** | **1** | **2** | **3** | **4** | **5** | **6** | **7** | **8** | **9** | **10** | **11** | **12** | **13** | **14** | ***FINAL QUADAS***  ***SCORE*** |
| --- | --- | --- | --- | --- | --- | --- | --- | --- | --- | --- | --- | --- | --- | --- | --- |
| **ter Horst et al. 2009** | x | x |  | x |  |  |  | x | x | x | x |  | x | x | 9 |
| **Bourgeois et al 2008** | x | x |  | x |  |  |  | x | x |  |  | x | x |  | 7 |
| **Antinori et al. 2007** | x | x | x | x |  |  |  | x | x |  |  | x |  |  | 7 |
| **Goswami et al. 2007** | x |  | x | x | x |  | x | x | x |  |  |  |  |  | 7 |
| **Sinha et al. 2006** | x |  | x | x |  |  | x | x | x |  |  |  |  |  | 6 |
| **Bossolasco et al. 2003** | x | x | x | x | x | x | x |  |  |  |  | x | x | x | 10 |
| **Cruz et al. 2002** | x | x | x | x | x | x | x | x | x |  |  | x | x | x | 12 |
| **Fisa et al. 2002** | x | x | x | x | x | x |  | x | x |  |  | x | x |  | 10 |
| **Hailu et al 2002** | x | x | x | x | x |  |  | x | x | x |  | x | x | x | 11 |
| **Campino et al. 2000** | x | x | x | x | x | x |  | x | x |  |  |  |  |  | 8 |
| **Moreno et al. 2000** | x | x | x | x | x | x | x | x | x |  |  |  | x |  | 10 |
| **Hofman et al. 2000** | x | x | x | x | x | x | x | x | x |  |  |  | x | x | 11 |
| **Santos-Gomes et al. 2000** | x | x | x | x | x |  |  | x | x |  |  |  |  | x | 9 |
| **Medrano et al. 1998** | x |  | x | x | x | x | x | x | x |  |  | x | x | x | 11 |
| **Houghton et al. 1998** | x |  | x | x | x | x | x | x | x |  |  |  | x |  | 9 |
| **Kubar et al. 1998** | x | x | x | x |  |  | x | x | x |  |  |  | x | x | 9 |
| **Agostoni et al.1998** | x | x | x | x | x | x | x | x | x |  |  | x | x | x | 12 |
| **Costa et al. 1996** | x |  | x |  |  |  |  | x |  | x | x |  | x | x | 7 |
| **Gasser et al. 1996** | x |  | x | x | x |  |  | x | x | x | x |  |  |  | 8 |
| **Nigro et al. 1996** | x | x | x | x |  |  |  | x | x |  |  |  | x | x | 8 |
| **Piarroux et al. 1996** | x | x | x | x |  |  |  | x | x |  |  |  | x | x | 8 |
| **Gallardo et al. 1996** | x | x | x | x | x | x | x | x |  |  |  |  | x | x | 10 |
| **Cardeñosa et al. 1996** | x |  | x |  | x |  | x | x | x |  |  |  |  |  | 6 |
| **Rosenthal et al. 1995** | x | x | x | x | x | x | x | x | x |  |  |  | x | x | 11 |
| **Ribera et al. 1995** | x | x | x | x | x | x | x | x | x |  |  | x | x | x | 12 |
| **Lopez-Velez et al. 1995** | x | x | x | x | x | x | x | x | x |  |  |  | x |  | 10 |
| **Daleine et al. 1994** | x |  |  |  |  |  | x | x |  |  |  |  |  |  | 3 |
| **Hernandez et al. 1993** | x |  | x | x |  |  | x | x | x |  |  |  |  |  | 6 |
| **Gradoni et al. 1993** | x |  | x | x | x |  | x | x |  |  |  |  |  |  | 6 |
| **Mary et al. 1992** | x | x | x | x | x | x | x | x | x |  |  | x |  |  | 10 |
| **del Mar et al. 1991** | x | x | x | x | x | x | x | x | x |  |  |  |  |  | 9 |
| **Montalban et al. 1990** | x | x | x | x | x |  |  | x | x |  |  |  | x |  | 8 |
| **Berenguer et al. 1989** | x | x | x | x | x | x | x | x | x |  |  |  | x |  | 10 |
