# Supplementary material for: The Diagnostic Accuracy of Serologic and Molecular Methods for Detecting Visceral Leishmaniasis in HIV Infected Patients: Meta-Analysis
Source: PLoS Negl Trop Dis. 2012 May 29;6(5):e1665. doi: 10.1371/journal.pntd.0001665 (PMC3362615; doi:10.1371/journal.pntd.0001665)
Supplement: Table S3 — Individual performance of studies evaluating serological tests. (DOC) [file pntd.0001665.s003.doc]

Table S3- Individual performance of studies evaluating serological tests

| IFAT | | | | | | | | |
| --- | --- | --- | --- | --- | --- | --- | --- | --- |
| **Reference** | **Country** | **True positive** | **False positive** | | **False Negative** | **True negative** | **Sensitivity**  **95% Confidence interval** | **Specificity**  **95% Confidence interval** |
| **Antinori et al. 2007** | Italy | 11 |  | | 7 |  | 0.611  0.386-0.797 |  |
| **Bossolasco et al. 2003** | Italy | 6 |  | | 3 |  | 0.667  0.354-0.879 |  |
| **Cruz et al. 2002** | Spain | 19 |  | | 19 |  | 0,500  0.348-0.651 |  |
| **Moreno et al. 2000** | Spain | 8 |  | | 9 |  | 0.471  0.262-0.690 |  |
| **Hofman et al.**  **2000** | France | 9 |  | | 5 |  | 0.643  0.388-0837 |  |
| **Medrano et al. 1998** | Spain | 2 | 0 | | 16 | 14 | 0.111  0,031-0,328 | 1  0.785-1.000 |
| **Agostoni et al. 1998** | Italy | 9 |  | | 3 |  | 0.750  0.468-0.911 |  |
| **Houghton et al. 1998** | Italy | 30 |  | | 26 |  | 0.536  0.407-0.659 |  |
| **Gasser et al. 1996** | Spain | 7 |  | | 7 |  | 0.500  0.268-0.732 |  |
| **Nigro et al. 1996** | Italy | 6 | 8 | | 3 | 83 | 0.667  0.354-0.879 | 0.912  0.836-0.955 |
| **Gallardo et al. 1996** | Spain | 1 | 1 | | 6 | 104 | 0.143  0.007- 0.513 | 0.990  0.948-0.995 |
| **Cardeñosa et al. 1996** | Spain | 11 |  | | 4 |  | 0.733  0.480-0.891 |  |
| **Piarroux et al. 1996** | Spain | 13 |  | | 11 |  | 0.541  0.351-0.721 |  |
| **Rosenthal et al. 1995** | France | 26 |  | | 21 |  | 0.553  0.412-0.686 |  |
| **Lopez-Velez et al. 1995** | Spain | 8 |  | | 16 |  | 0.333  0.179-0.533 |  |
| **Ribera et al. 1995** | Spain | 4 |  | | 12 |  | 0.250  0.102-0.495 |  |
| **Daleine et al. 1994** | France | 11 |  | | 5 |  | 0.687  0.444-0.858 |  |
| **Hernadez et al. 1993** | Spain | 6 | 21 | | 6 | 77 | 0,500  0.254-0.746 | 0.786  0.694-0.855 |
| **Gradoni et al. 1993** | Italy | 18 |  | | 4 |  | 0.818  0.615-0.927 |  |
| **Montalban et al. 1990** | Spain | 11 |  | | 18 |  | 0.379  0.227-0.56 |  |
| **Berenguer et al. 1989** | Spain | 2 |  | | 7 |  | 0.222  0.063-0.547 |  |
| **ELISA** | | | | | | | | |
| **Fisa et al. 2002** | Spain |  | 2 |  | | 26 |  | 0.928  0.773-0.980 |
| **Medrano et al. 1998** | Spain | 4 | 3 | 16 | | 11 | 0.200  0.081-0.416 | 0.786  0.524-0.924 |
| **Houghton et al. 1998** | Italy | 46 | 0 | 10 | | 12 | 0.821  0.702-0.900 | 1  0.758-1.000 |
| **Piarroux et al. 1996** | Spain | 14 |  | 10 | |  | 0.583  0.388-0.755 |  |
| **Rosenthal et al. 1995** | France | 26 |  | 21 | |  | 0,553  0.412-0.686 |  |
| **Daleine et al. 1994** | France | 16 |  | 0 | |  | 1  0.806-1 |  |
| **BLOT** | | | | | | | | |
| **Fisa et al. 2002** | Spain |  | 4 |  | | 24 |  | 0.857  0.685-0.943 |
| **Moreno et al. 2000** | Spain | 11 |  | 2 | |  | 0.846  0.578-0.957 |  |
| **Santos-Gomes et al. 2000** | Portugal | 14 | 8 | 2 | | 11 | 0.875  0.639-0.965 | 0.578  0.363-0.769 |
| **Kubar et al. 1998** | France | 13 | 15 | 1 | | 207 | 0.928  0.685-0.996 | 0.932  0.892-0.959 |
| **Medrano et al. 1998** | Spain | 14 | 2 | 4 | | 12 | 0.778  0.548-0.91 | 0.857  0.600-0.959 |
| **Cardeñosa et al. 1996** | Spain | 14 |  | 1 | |  | 0.933  0.702-0.997 |  |
| **Piarroux et al. 1996** | Spain | 15 |  | 9 | |  | 0,625  0.427-0.788 |  |
| **Rosenthal et al. 1995** | France | 15 |  | 3 | |  | 0.833  0.608-0.942 |  |
| **Mary et al. 1992** | France | 11 |  | 0 | |  | 1  0.741-1 |  |
| **DAT** | | | | | | | | |
| **ter Horst et al. 2009** | Ethiopia | 39 |  | 5 | |  | 0.886  0.760-0.950 |  |
| **Sinha et al. 2006** | India | 6 | 0 | 2 | | 10 | 0,750  0.409-0.928 | 1  0.722-1.000 |
| **Hailu et al. 2002** | Ethiopia | 46 | 3 | 2 | | 4 | 0.958  0.860-0.988 | 0.571  0.250- 0.842 |
| **Nigro et al. 1996** | Italy | 4 | 2 | 5 | | 89 | 0.444  0.189-0.733 | 0.978  0.923-0.994 |
| **RECOMBINANT K39 DIPSTICK TEST (IMMUNOCHROMATOGRAPHIC TEST)** | | | | | | | | |
| ter Horst et al. 2009 | Ethiopia | 34 |  | 10 | |  | 0.773  0.630-0.872 |  |
| Goswami et al. 2007 | India | 12 | 0 | 0 | | 60 | 1  0.757-1 | 1  0.939-1 |
