# Supplementary material for: The Diagnostic Accuracy of Serologic and Molecular Methods for Detecting Visceral Leishmaniasis in HIV Infected Patients: Meta-Analysis
Source: PLoS Negl Trop Dis. 2012 May 29;6(5):e1665. doi: 10.1371/journal.pntd.0001665 (PMC3362615; doi:10.1371/journal.pntd.0001665)
Supplement: Table S4 — Individual performance of studies evaluating molecular tests. Footnote: polimerase chain reaction (PCR). (DOC) [file pntd.0001665.s004.doc]

Table S4- Individual performance of studies evaluating molecular tests

| PCR (BONE MARROW) | | | | | | | | | |
| --- | --- | --- | --- | --- | --- | --- | --- | --- | --- |
| **Reference** | **Country** | | **True positive** | **False positive** | **False Negative** | **True negative** | **Sensitivity**  **95% Confidence interval** | | **Specificity**  **95% Confidence interval** |
| Bourgeois et al. 2008* | France | | 27 |  | 0 |  | 1  0.875-1 | |  |
| Antinori et al. 2007* | Italy | | 13 |  | 1 |  | 0,928  0.685-0.996 | |  |
| Cruz et al. 2002 | Spain | | 38 |  | 0 |  | 1  0.908-1 | |  |
| **PCR (PERIPHERAL BLOOD)** | | | | | | | | | |
| Bourgeois et al. 2008 | | France | 27 |  | 0 |  | | 1  0.875-1 |  |
| Antinori et al. 2007 | | Italy | 20 |  | 0 |  | | 1  0.839-1 |  |
| Bossolasco et al 2003 | | Italy | 10 | 0 | 0 | 15 | | 1  0.722-1.000 | 1  0.796-1.000 |
| Cruz et al. 2002 | | Spain | 34 |  | 4 |  | | 0,895  0.759-0.958 |  |
| Fisa et al. 2002 | | Spain | 13 | 5 | 0 | 23 | | 1  0.772-1.000 | 0.821  0.644-0.921 |
| Campino et al. 2000 | | Portugal | 13 |  | 5 |  | | 0,722222  0.491-0.875 |  |
| Costa et al. 1996 | | France | 12 | 0 | 1 | 77 | | 0.923  0.667-0.996 | 1  0.952-1.000 |
| Piarroux et al. 1996 | | Spain | 19 |  | 6 |  | | 0,76  0.566-0.885 |  |
